# On the quest of reliable 3D dynamic *in vitro* blood-brain barrier models using polymer hollow fiber membranes: Pitfalls, progress, and future perspectives

**DOI:** 10.3389/fbioe.2022.1056162

**Published:** 2022-11-22

**Authors:** Marián Mantecón-Oria, María J. Rivero, Nazely Diban, Ane Urtiaga

**Affiliations:** ^1^ Departamento de Ingenierias Química y Biomolecular, Universidad de Cantabria, Santander, Spain; ^2^ Instituto Marqués de Valdecilla (IDIVAL), Santander, Spain

**Keywords:** blood-brain barrier (BBB), dynamic *in vitro* (DIV)-BBB models, hollow fiber polymer membranes, microstructural properties, perfusion

## Abstract

With the increasing concern of neurodegenerative diseases, the development of new therapies and effective pharmaceuticals targeted to central nervous system (CNS) illnesses is crucial for ensuring social and economic sustainability in an ageing world. Unfortunately, many promising treatments at the initial stages of the pharmaceutical development process, that is at the *in vitro* screening stages, do not finally show the expected results at the clinical level due to their inability to cross the human blood-brain barrier (BBB), highlighting the inefficiency of *in vitro* BBB models to recapitulate the real functionality of the human BBB. In the last decades research has focused on the development of *in vitro* BBB models from basic 2D monolayer cultures to 3D cell co-cultures employing different system configurations. Particularly, the use of polymeric hollow fiber membranes (HFs) as scaffolds plays a key role in perfusing 3D dynamic *in vitro* BBB (DIV-BBB) models. Their incorporation into a perfusion bioreactor system may potentially enhance the vascularization and oxygenation of 3D cell cultures improving cell communication and the exchange of nutrients and metabolites through the microporous membranes. The quest for developing a benchmark 3D dynamic *in vitro* blood brain barrier model requires the critical assessment of the different aspects that limits the technology. This article will focus on identifying the advantages and main limitations of the HFs in terms of polymer materials, microscopic porous morphology, and other practical issues that play an important role to adequately mimic the physiological environment and recapitulate BBB architecture. Based on this study, we consider that future strategic advances of this technology to become fully implemented as a gold standard DIV-BBB model will require the exploration of novel polymers and/or composite materials, and the optimization of the morphology of the membranes towards thinner HFs (<50 μm) with higher porosities and surface pore sizes of 1–2 µm to facilitate the intercommunication *via* regulatory factors between the cell co-culture models of the BBB.

## 1 Introduction

**FIGURE 1:**
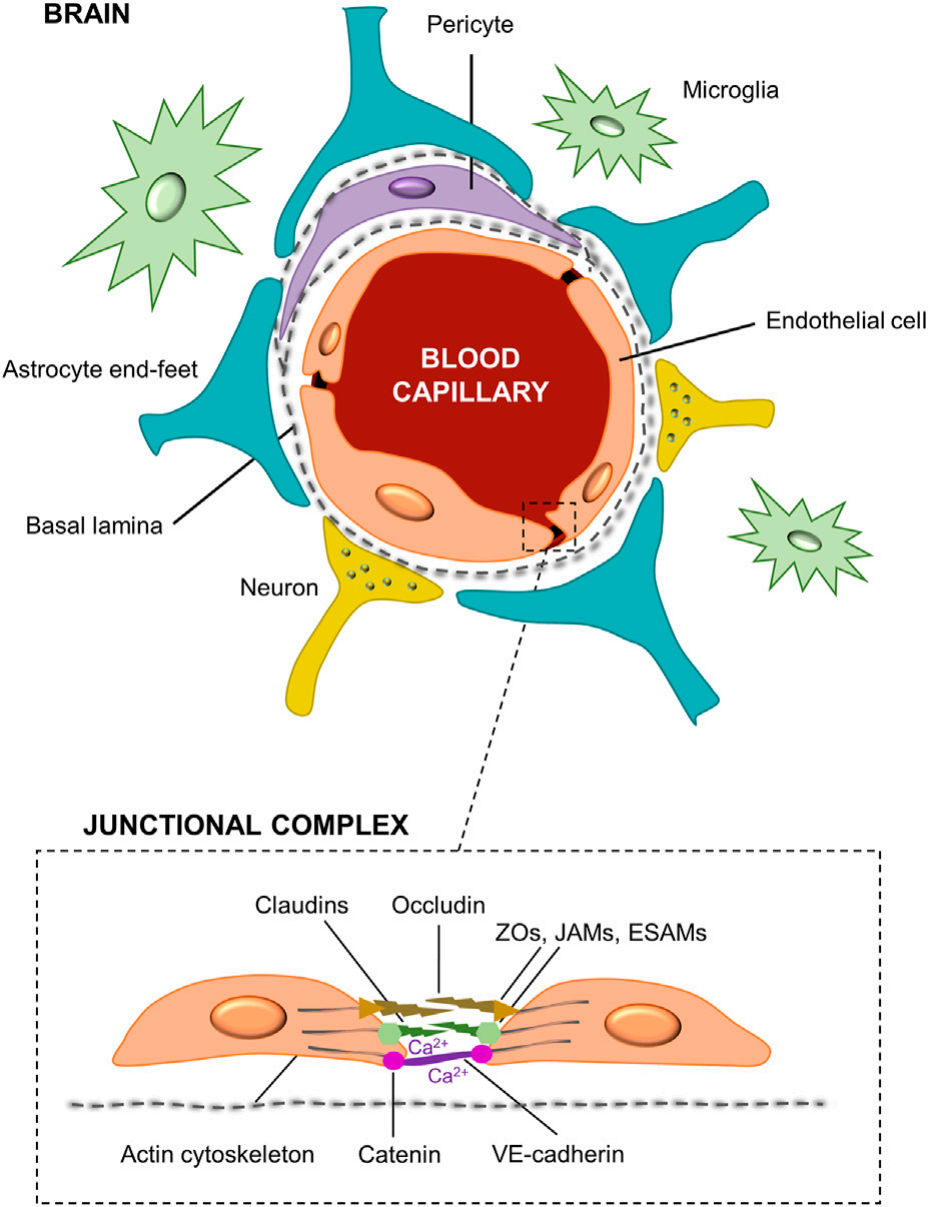
Structural diagram of the blood-brain barrier and its cellular constituents. The BBB is formed by capillary endothelial cells surrounded by a basement membrane and astrocytes end-feet. It is also constituted by neurons, pericytes and microglia cells. A detail of the brain endothelial cells unions, i.e., tight and adherens junctions, namely as junctional complex is presented.

In recent years, the percentage of patients that suffer from neurological diseases is getting higher, being recognized as one of the major causes of death and disability worldwide (Feigin, 2019). Thus, the development of new therapies and effective drug pharmaceuticals targeted to Central Nervous System (CNS) illnesses is critical (He et al., 2014; Di and Kerns, 2015). The blood-brain barrier (BBB), which is a dynamic and complex structure that separates the CNS from the circulatory system, plays an important role in the understanding of CNS physiology and pharmacokinetic studies (Stanness et al., 1997). It is constituted by different types of cells as can be seen in Figure 1. Among them, the most important are the human brain microvascular endothelial cells (HBMECs), linked by complex tight junctions, that regulates the exchange of nutrients, oxygen, ions, and metabolites between the circulating blood (apical side) and the extracellular fluids of the nervous tissue (basolateral side) in the CNS (Mantecón-Oria et al., 2020). The endothelial cells are surrounded by a basement membrane and astrocytic perivascular end-feet, which provide architectural support for neurons. Figure 1 also shows microglia cells and pericytes. Particularly, some studies have suggested that astrocytes play a key role in the development and function of the brain by regulating the phenotype of endothelial cells through the control of cell–cell communication *via* soluble factors (Chandrasekaran et al., 2016). Among these soluble factors, the transforming growth factor-β (TGFβ), the glial-derived neurotrophic factor (GDNF), and the basic fibroblast growth factor (bFGF) can induce BBB endothelial cells phenotype to enhance *in vitro* BBB reconstruction (Abbott et al., 2006). Moreover, astrocytes regulate the exchange of molecules in and out from the brain that control and modulate the neurotoxic effects, and maintain brain homeostasis (Peng et al., 2013; Daneman and Prat, 2015). This restrictive barrier is achieved by the high expression of tight junctional proteins like claudins (e.g., claudin-3, claudin-5) and cytoplasmatic occludens proteins (ZOs), as well as the junctional and the endothelial selective adhesion molecules (JAMs and ESAMs, respectively), involved in the formation and maintenance of the tight junctions (Abbott et al., 2006). Additionally, the Ca^2+^-dependent serine protein kinase (CASK) acts as a second messenger for BBB regulation activity binding the intramembrane proteins. Parallelly, tight junctions are intermingled with the adherens junctions in BBB endothelial cells. These adherens junctions, located more basally, are composed by catenins and cadherins proteins contributing to the barrier function and connecting the actin cytoskeleton. Among them, the most important is the vascular endothelial cadherin (VE-cadherin) (Long et al., 2022). The molecular composition of the endothelial tight junctions is also presented in Figure 1. The restrictive mass transport of BBB is essential for the control of CNS homeostasis of the brain microenvironment, neuronal function and activity, and for the isolation of the nervous tissue from potentially noxious substances as toxins or pathogens (Santaguida et al., 2006; Zhao et al., 2021).

**FIGURE 2:**
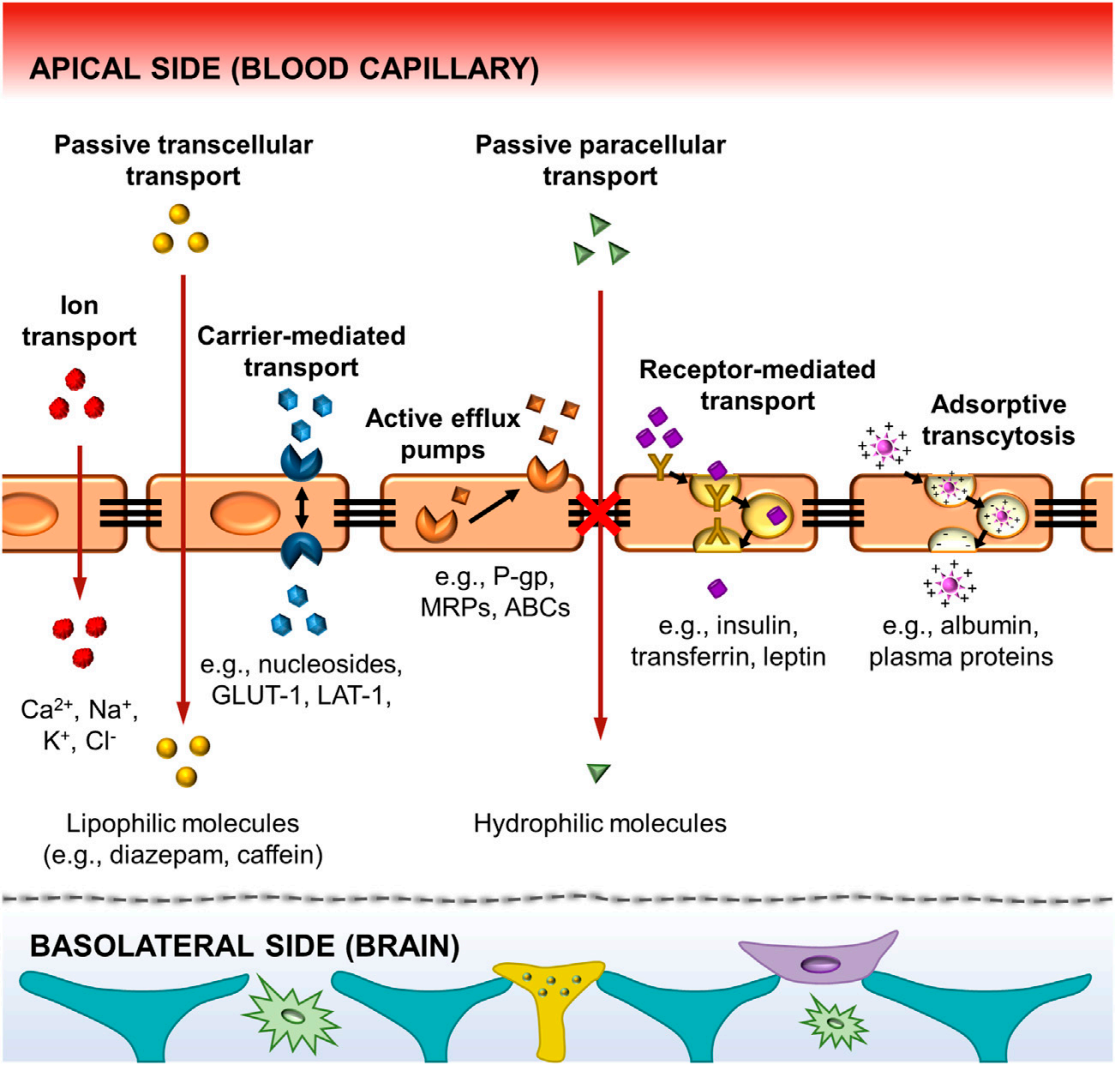
Schematic illustration of the principal pathways for molecular traffic across the BBB. It includes the active transport by the carriers-mediated, the efflux pumps, the receptors-mediated and the adsorptive transcytosis. The passive transport includes the transcellular diffusion of lipophilic molecules across the BBB, and the paracellular transport of hydrophilic molecules. The tight junctions between the endothelial cells prevent several molecules from easily crossing the BBB. Finally, ion transport is achieved by ion channels. Some representative molecular substances following each type of mechanism are also presented in the scheme.

Apart from BBB structure, it is important to know how the routes of the substances across the BBB and its regulation (Figure 2). Active and passive transcellular transport are the main mechanisms by which the molecules can enter the brain through the BBB. The active transcellular transport includes mass transport mechanisms such as carrier-mediated transport, efflux pumps, receptor-mediated transport, and adsorptive transcytosis (Di and Kerns, 2015; Curley and Cady, 2018). The carrier-mediated transport enables different molecules such as glucose (GLUTs), nucleosides, and large neutral amino acid transporters (LATs) to cross the cell membrane to the brain *via* substrate-specific transporters. Remarkably, GLUT-1 and LAT-1 transporters are bidirectional moving in or out of the endothelial cells by the concentration gradient, being the GLUT-1 transporter the primary source of energy for the brain (Sweeney et al., 2019). A battery of ATP-driven drug efflux pumps, i.e., ATP-binding cassette transporters (ABCs) such as P-glycoprotein (P-gp), breast cancer resistance proteins (BCRPs), and multidrug resistance proteins (MRPs), prevents brain accumulation of drugs and xenobiotics *via* active efflux of these compounds from the brain or endothelial cells to the blood (Wong et al., 2013; Long et al., 2022). Then, some lipoproteins as insulin and transferrin penetrate the brain through receptors-mediated transcytosis. Otherwise, some plasma proteins can be transported by adsorptive transcytosis due to the presence of cations in their chemical structure. In passive mechanisms, the transcellular transport supports the diffusion of small lipid-soluble agents through the large surface area of the lipid membranes of the endothelial cells. Interestingly, drug delivery of organophilic molecules across the BBB mainly follows a transcellular lipophilic pathway (Abbott et al., 2006). In contrast, water-soluble molecules such as polar drugs should follow a paracellular transport, which is impeded by the presence of tight junctions. Finally, small ions (Ca^2+^, Na^+^, K^+^, Cl^−^) can cross the BBB through ion channels (Wong et al., 2013). All these mechanisms are represented in Figure 2. More information regarding relevant aspects of the structure and regulation mechanisms of the blood-brain barrier were further expanded in other review articles (Di and Kerns, 2015; Hladky and Barrand, 2016; Sweeney et al., 2019; Lochhead et al., 2020; Gosselet et al., 2021).

It is widely reported that BBB dysfunction and the loss of structural integrity leads to the progression of a great number of neurological diseases such as Alzheimer, Parkinson, epilepsy, multiple sclerosis, and brain cancer (Stanness et al., 1997; Cucullo et al., 2008; Chen et al., 2021). For instance, the multiple sclerosis as chronic neuropathology involves an early step of BBB breakdown with the downregulation of laminin in the basement membrane and selective loss of claudin-1 and claudin-3 that precedes neuronal damage (Abbott et al., 2006). In contrast, the Parkinson’s disease is consistent with the dysfunction of the BBB by the reduction of the P-gp efficacy. Unfortunately, most of the promising therapeutic approaches to combat the neurological diseases fail to show the expected results since most of the drugs are not able to cross the BBB representing a critical hurdle for diseases treatment. The BBB is so effective that nearly the 100% of large molecule neurotherapeutics and more than the 98% of small molecule drugs are blocked from entering the brain, also preventing the use of imaging techniques for the diagnosis of neurodegenerative diseases (Pardridge, 2005). Therefore, the understanding of the human brain physiology and the mechanisms of drug administration and liberation, is essential to create and optimize new methods aiming at the opening of the BBB to improve drug permeability, and for the comprehension of the progression of CNS illnesses (Stanness et al., 1997; Neuhaus et al., 2006; Ferber, 2007).

Over the last years, advances in the development of novel functional materials and nanotechnology for tissue engineering (TE) aim at contributing to the study and mitigation of the effects of neurodegenerative diseases in an ageing world. TE approach relies on cellular seeding and proliferation supported in scaffolds, i.e., biopolymeric membranes with specific features, that ultimately produce an engineered organ or tissue constructs (Diban and Stamatialis, 2014; Diban et al., 2017; Morelli et al., 2021; Cheng et al., 2022). These biopolymeric membranes can then be implemented into a perfusion bioreactor that provides mechanical stimuli, mass transfer and oxygenation in 3D cell cultures (Diban et al., 2018). Because of the complexity of the *in vivo* BBB structure, simplified *in vitro* BBB models have been used to gain knowledge into designing biochemical strategies to allow the temporarily opening of the BBB for the efficient delivery of drugs to the CNS, and saving costs in pre-screening and experimental studies (Santaguida et al., 2006; Mahaffey, 2012; He et al., 2014). The assessment of the BBB formation and its integrity in engineered *in vitro* models consider different structural, microenvironmental and functional aspects that were previously reported (Bhalerao et al., 2020; Williams-Medina et al., 2021; Jagtiani et al., 2022). These include the formation of tight cellular junctions (Machado, 2012; Banerjee et al., 2016; Destefano et al., 2018), and the expression of BBB markers such as von Willebrand Factor (vWF) and the protein cluster of endothelial cell differentiation 31 (CD31) (Löscher and Potschka, 2005; Gil-Martins et al., 2020). The use of super-resolution imaging techniques as environmental scanning electron microscope (ESEM), structured illumination microscopy (SIM), stimulated emission depletion (STED) microscopy, and single molecule localization microscopy (SMLM) enables imaging tight and adherens junctions at the nanoscale, which appear as a network of contact points between extracellular claudins, occludins, and other transmembrane proteins (Destefano et al., 2018; Gonschior et al., 2020). In the same way, the cell viability and junction proteins expression can be evaluated by immunocytochemistry using light-phase/confocal imaging (Jagtiani et al., 2022). Moreover, the transendothelial electrical resistance (TEER), which is a non-invasive technique that measures the electrical impedance across the monolayer of endothelial cells (ECs) forming the BBB and its paracellular tightness, is continuously monitored (Srinivasan and Kolli, 2019; Neuhaus, 2021; Jagtiani et al., 2022). The transepithelial permeability and paracellular markers (e.g., dextran, mannose, sucrose, bovine serum albumin, immunoglobulin) are also evaluated (Natarajan et al., 2017; Bhalerao et al., 2020; Williams-Medina et al., 2021), as well as the cellular metabolism as nutrients consumption and metabolites production (Janigro et al., 2000; Cucullo et al., 2002), and the microenvironmental conditions (e.g., extracellular matrix (ECM), shear stress and cell sources) (Bischel et al., 2016; Linville and Searson, 2021). Specifically, the wall shear stress in 3D models can be determined by particle imaging velocimetry, analyzing how fluorescent beads move in the perfusion culture media, or by calculating the flow rate in the system (Destefano et al., 2018). The dynamic monitorization of some of the parameters mentioned above can serve as benchmark to validate the *in vitro* BBB model formation.

This article will be focused on unveiling the state-of-art of the *in vitro* BBB models most used nowadays: 1) Transwell inserts as static model, and 2) microfluidic devices and flow-based hollow fiber systems as dynamic models. Particularly, we will analyze the use of dynamic perfusion bioreactor systems that utilize polymeric hollow fiber membranes (HFs) as scaffolds for successfully mimicking the physiological environment, and to gain insights into the influence of different HFs properties, such as the polymeric materials used, the morphological features (pore size, porosity, thickness … ) and other performing properties (permeability, nutrients transport, TEER measurements … ), for the development of reliable 3D dynamic *in vitro* (DIV)-BBB models. Moreover, the main benefits and downsides of using hollow fiber perfusion bioreactors for DIV-BBB models will be collected, and some guidelines will be hinted as the main novelty of this work to help further investigations and direct future strategic advances in the design of 3D DIV-BBB flow-based hollow fiber models.

## *2 In vitro* BBB models overview

### 2.1 Static cell culture models

**FIGURE 3:**
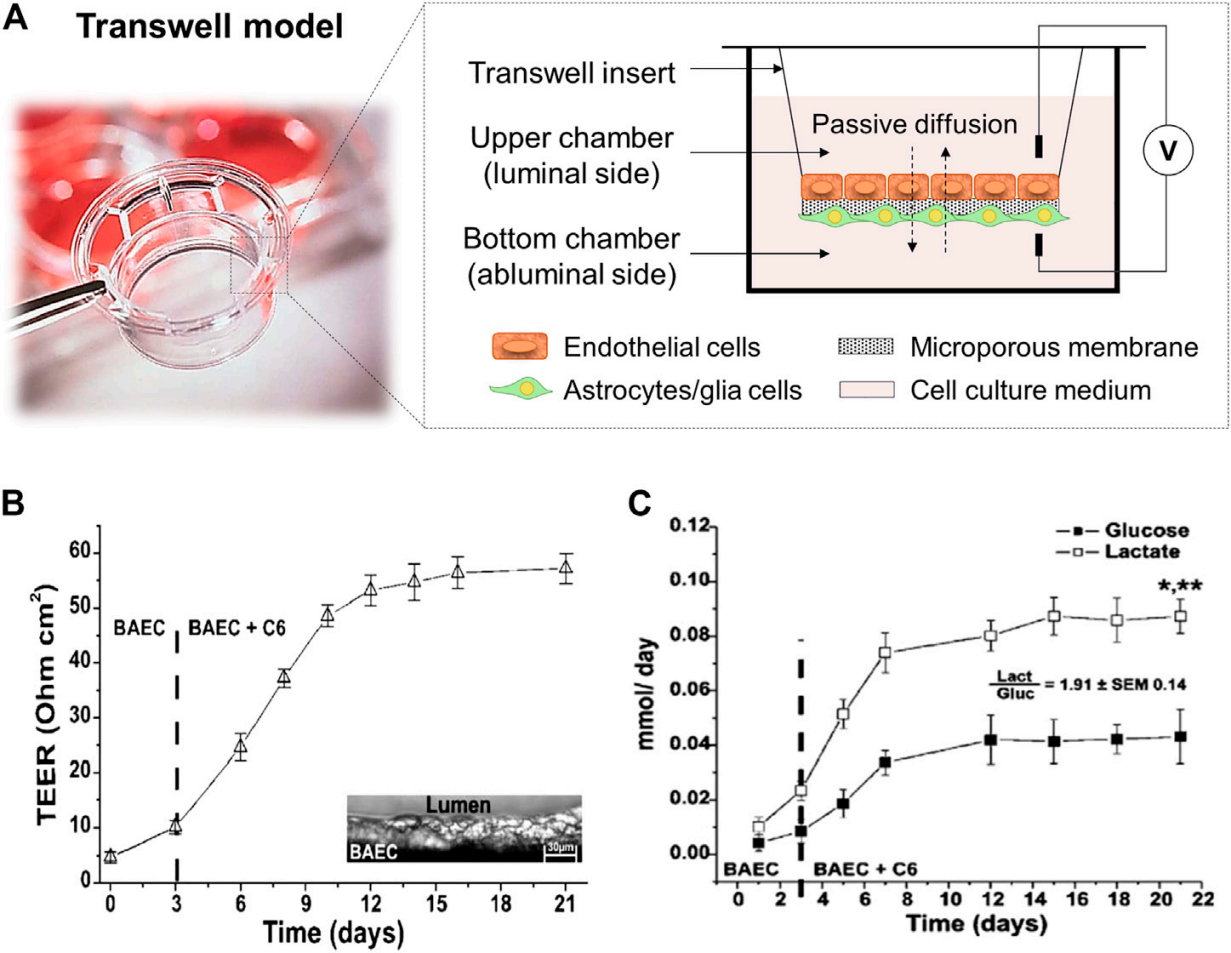
**(A)** Scheme of a Transwell cell coculture contact model divided by a flat microporous membrane. ECs are seeded on the luminal side of the membrane (upper chamber) and astrocytes on the abluminal side (bottom chamber). The diffusion of compounds occurs side-by-side across the membrane. It is presented a picture of a Corning™ Transwell™ multiple well plate with permeable polyester membrane inserts provided by Fisher Scientific (Thermo Fisher Scientific, 2022). **(B)** TEER values achieved in a Transwell coculture model of BAEC and C6 glia cells for 21 days, and **(C)** cell metabolism expressed through glucose consumption (mmol/day) and lactate production (mmol/day). The ratio between the lactate production and the glucose consumption is around two demonstrating an anaerobic metabolic pathway for cells cultured under static conditions. *, *p* < 0.05 refers to a statistically significant metabolic increase and **, *p* < 0.05 represents the statistically significant differences between glucose consumption and lactate production. Both graphs has been reprinted with permission from (Santaguida et al., 2006).

The development of *in vitro* BBB models was initiated with static cell monolayer cultures and 2D static cocultures in flat Transwell systems (DeBault and Cancilla, 1980; Audus and Borchardt, 1986; Dehouck et al., 1994; Daniels et al., 2013), and has evolved over time. Transwell systems (Figure 3A) comprised two compartments separated by a semipermeable plastic membrane in which vertical diffusion occurs. This basic *in vitro* BBB culture model involves the growth of a monolayer of ECs in the luminal side of the Transwell flat insert membrane, which represents the “capillary blood”, whereas the well in which it is inserted represents the “brain” or abluminal side where astroglia cells are cultured (Jagtiani et al., 2022). In these Transwell systems, endothelial and astroglia cells can be in contact or not. In the noncontact models, astroglia cells grow on the bottom of the culture plate, whereas in the contact models, they grow in the opposite side of the Transwell membrane (Cai et al., 2021).

Different semipermeable membrane materials have been studied in Transwell devices. In 1994, Dehouck et al. (1994) reported the ability of rat astrocytes, cultured in plate membrane inserts (Millicell-CM, Millipore Corp.) made of hydrophilic polytetrafluoroethylene (PTFE) of 0.4 µm pore size, to modulate bovine brain capillary endothelial cells expression. Other similar works used Transwell filters (Corning Inc.) with 0.4 µm pore size membrane inserts made of polycarbonate (PC) (McAllister et al., 2001), or polyester (PE) (Perrière et al., 2007). Afterwards, Daniels et al. (2013), used PTFE Transwell inserts (BD Falcon) with 3 µm pore size for trans-BBB immune migration assays improving drugs passage. These models provide a simply, low-cost, and well-established methodology able to replicate confluent monolayer of cells, and basic cell cocultures to allow preliminary studies of transporter kinetics, permeability, and drug screening (Perrière et al., 2005; Perrière et al., 2007; Yao and Tsirka, 2011). Moreover, a rapid and non-destructive measurement of TEER can be achieved with values around 150 Ω cm^2^, sufficient to perform drug permeability studies (He et al., 2014; Neuhaus, 2021). For instance, Santaguida et al. (2006) studied a cell coculture model of bovine aortic endothelial (BAEC) and C6 rat glial tumor cell line differentiated towards astrocytes using traditional Transwell inserts with a PE membrane and analyzed important parameters, e.g., TEER values, glucose consumption and lactate production, sucrose paracellular marker and phenytoin drug permeability. The values achieved for TEER measurements were around 60 Ω cm^2^ (Figure 3B). In terms of cell metabolism, lactate production was 2-folding the glucose consumption, indicating the preferential anaerobic metabolism of cells (Figure 3C). In overall, these observations showed that in Transwell models the tissue oxygenation was compromised and moreover, with these membrane inserts, it is impossible to reach TEER values close to *in vivo* ones, e.g., 1,200 Ω cm^2^ for brain arterial vessels (Banerjee et al., 2016), and 5,000 Ω cm^2^ for human BBB (Srinivasan and Kolli, 2019). Furthermore, these systems provide 2D structures and static cell culture conditions which do not accurately mimic the *in vivo* microenvironment (e.g., lack of shear stress generated by the flow of blood in the lumen of the brain microvascular vessels), and exhibit several limitations such as low barrier tightness and efflux functionality, high cell membrane permeability, and lack of 3D cellular organization and cell-to-cell contact, which need to be overcome (Prashanth et al., 2021).

### 2.2 Dynamic cell culture models

To solve that issues, dynamic cell culture systems have raised to BBB models much closer to clinical relevance, and high throughput devices for better predictions and drug screening. Dynamic cell culture models introduce shear stress mimicking physiological conditions, which heavily affects the barrier and transport function of the BBB as well as the expression of tight junctions (He et al., 2014). Among these, there are two types that have received increasing attention during the last years: 1) microfluidic devices, and 2) DIV-BBB flow-based hollow fiber models.

**FIGURE 4:**
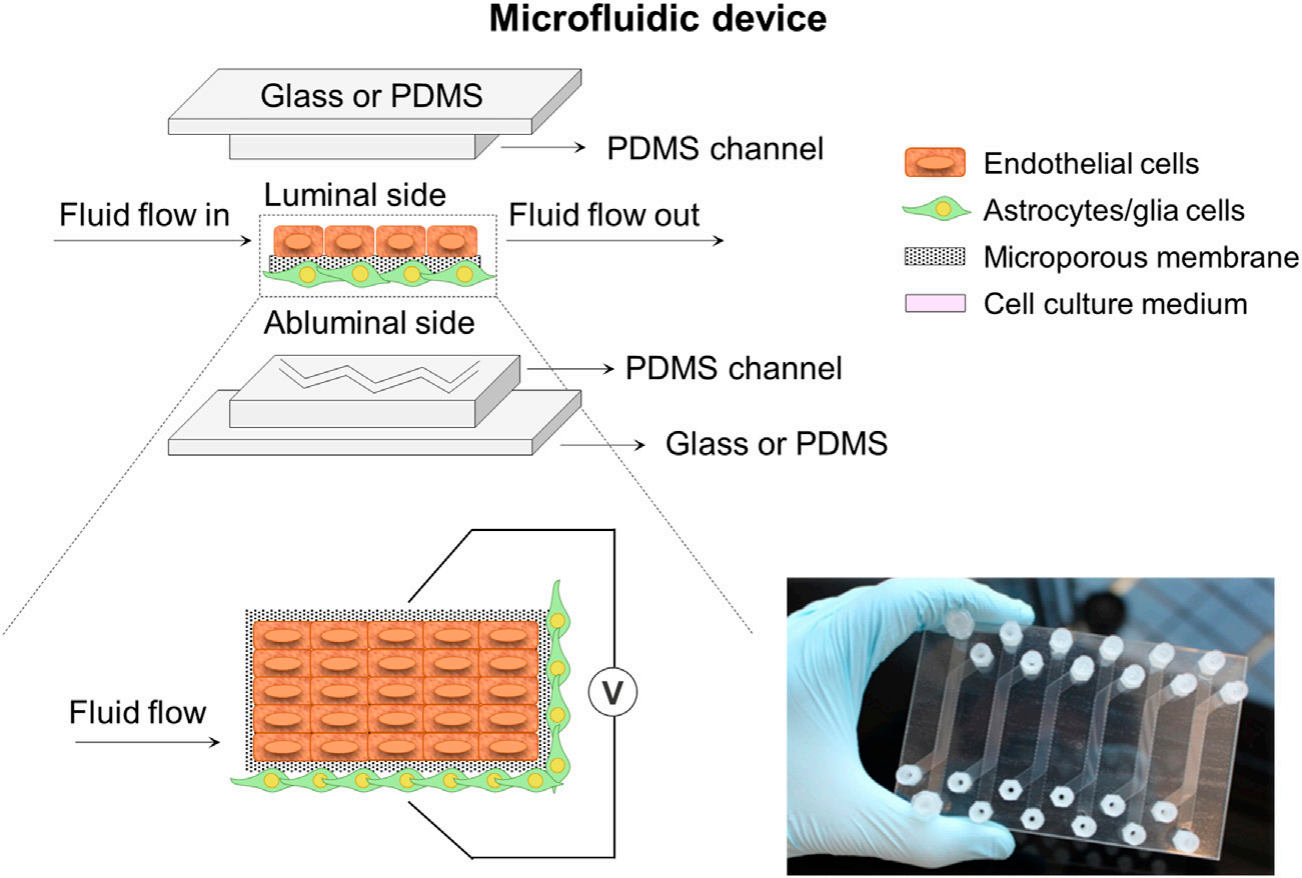
Schematic illustration of a microfluidic device system composed of two microchannels. These microchannels with specific patterns cross each other perpendicularly and are separated by a flat porous membrane to allow cell co-culture. The system could incorporate some electrodes to help with the TEER measurement at both sides of the membrane. A picture of a home-made microfluidic device in plate format configuration encompassing six conditions in parallel is also presented (Choublier et al., 2021).

Microfluidic miniaturized device models (Figure 4) comprise of two distinct compartments with specific patterned microchannels crossing each other perpendicularly that allow dynamic flow to create shear stress mimicking well the BBB environment, and enable the assessment of permeability (Nguyen et al., 2018; McNamara et al., 2021; Prashanth et al., 2021). A flat microporous membrane is located between the chambers to allow the co-culture of astrocytes and ECs by seeding them on either side of the membrane using flowing cell suspensions (Jagtiani et al., 2022). Choublier et al. (2021) used a polyethylene terephthalate (PET) semipermeable membrane with 0.4 µm pore size instead of a usual PC membrane observing a favorable differentiation of human cerebral microvascular endothelial (hCMEC/D3) cells. The microfluidic chips are usually fabricated by soft lithography, 3D printing or laser patterning techniques with several materials such as PC, polyetherimide (PEI), silicon, glass, and so on. However, the most commonly used material is polydimethylsiloxane (PDMS) (Mofazzal Jahromi et al., 2019), which is widely employed because of its low cost, easy fabrication, good optical transparency, high flexibility, gas permeability and relative biocompatibility (Hosic et al., 2021; Dai et al., 2022). However, these microfluidic models are still unsuitable for high-throughput studies due to the lack of standardized protocols for important parameters assessment and quantification, the complexity of technically demanding fabrication and its high costs (Jagtiani et al., 2022). Thus, microfluidic miniaturized device models require highly skilled research and laboratories for the design of a standard mold or protocol to obtain success in high-throughput microfluidic devices. Finally, as it happened in Transwell models, these systems exhibit several limitations in terms of recapitulating morphology, metabolism and expression profiles of cells as well as 3D cellular organization, working most of the studies in 2D configuration because of the intrinsic geometry of microfluidic devices (He et al., 2014; Coluccio et al., 2019; Pang et al., 2021). In this regard, many efforts have been driven to develop 3D extracellular matrix on microfluidic systems for specific applications, e.g., neural stem cells differentiation and regeneration, organoids-on-a-chip, and in brain, liver and cancer models (Wang et al., 2017; Mofazzal Jahromi et al., 2019; Zhao et al., 2021; Dai et al., 2022).

**FIGURE 5:**
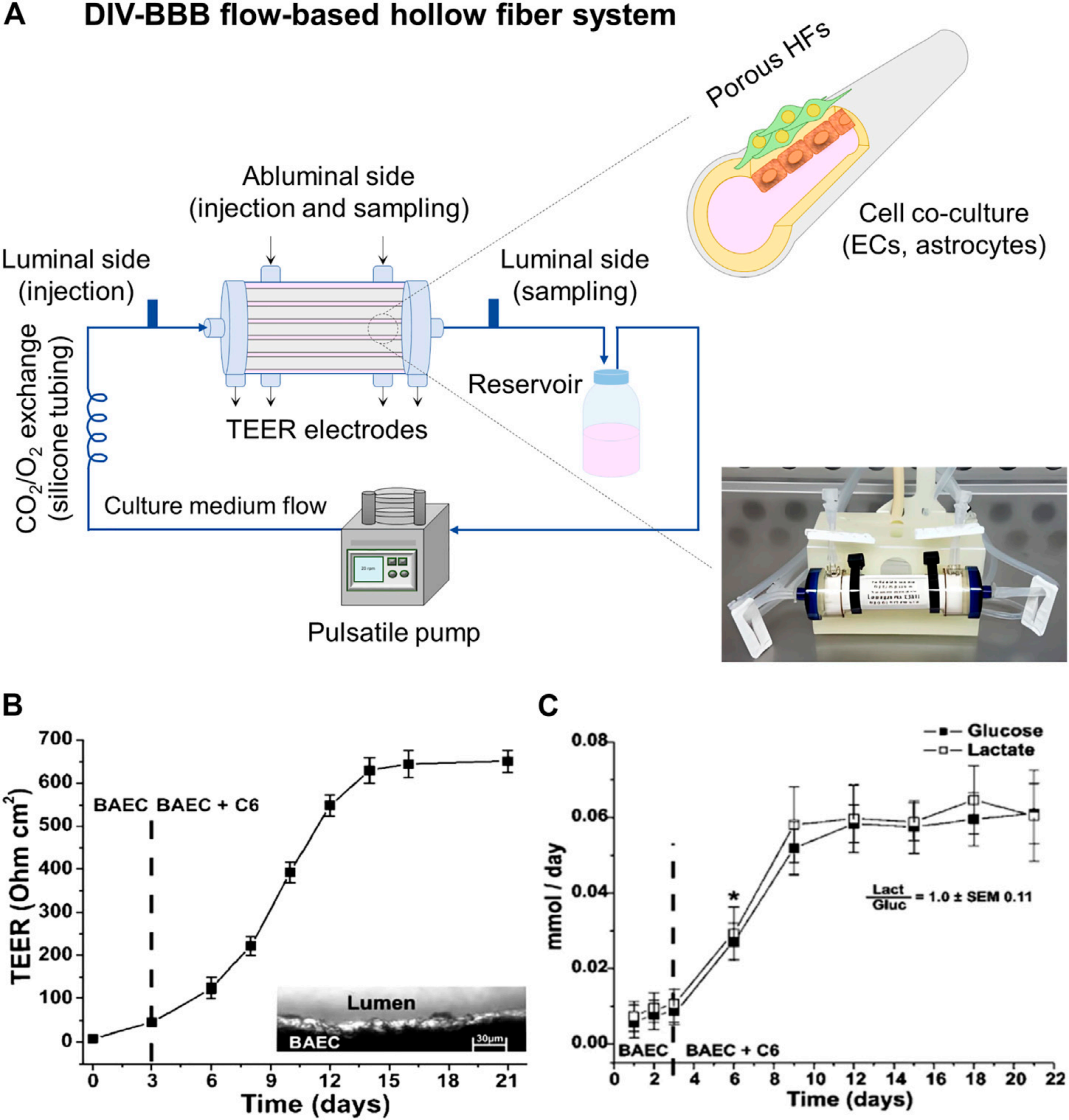
**(A)** DIV-BBB flow-based hollow fiber model, where ECs cells are cultured in the lumen of the HFs and astrocytes in the abluminal side. The HFs are sealed inside the perfusion bioreactor. The pulsatile pump uses silicone gas-permeable tubing to continuously supply fresh culture media in the perfusion bioreactor. It can be seen a picture of a commercial DIV-BBB module of medium size consisting of polysulfone HFs provided by Fiber Cell Systems (FiberCell Systems, 2022). **(B)** TEER values achieved in a 19 polypropylene HFs DIV-BBB model of BAEC and C6 glia cells for 21 days, and **(C)** cell metabolism expressed through glucose consumption (mmol/day) and lactate production (mmol/day). The ratio between the lactate production and the glucose consumption is around one demonstrating an aerobic metabolic pathway for cells cultured under dynamic conditions. *, *p* < 0.05 represents the statistically significant difference between glucose consumption and lactate production. Both graphs has been reprinted with permission from (Santaguida et al., 2006).

On the other hand, DIV-BBB flow-based hollow fiber models mainly consist of a dynamic perfusion bioreactor with a variable number of HFs (Figure 5A). The HFs act as capillary-like structures and permit 3D cell cocultures, supporting cell growth, proliferation, and differentiation until the *in vitro* reconstruction of the tissue (Santaguida et al., 2006). Therefore, these devices mimic the tubular vascular macrostructure and provides the tangential flow of the culture media through the lumen of the HFs with a soft perfusion or convective mass transport of nutrients, biomolecules, and regulatory factors through the membrane wall (Diban and Stamatialis, 2014; Diban et al., 2018). The HFs are encased in a housing shell creating two chambers: 1) the luminal side corresponding to the inner of the HFs, and 2) the outer space around the HFs named as abluminal side. The endothelial cells are cultured in the luminal side of the HFs developing a morphology close to the *in situ* 3D structures, while glia or neural cells, which provide differentiation factors enhancing BBB formation, are cultured in the external surface of the HFs (Santaguida et al., 2006; Cucullo et al., 2011). A media reservoir can be connected to the DIV-BBB system to pump the culture medium to the luminal and abluminal side of the HF perfusion bioreactor system, using a silicone gas-permeable tubing, which allows the exchange of oxygen and carbon dioxide (CO_2_) with the external environment before entering the HFs module (Figure 5A). The culture medium flows constantly through the lumen of the HFs, which ensures the mass transfer and exchange of nutrients (e.g., glucose and oxygen), the removal of metabolites produced (e.g., lactate and retinoic acid), and provides mechanical stimuli to the cells. This mechanical stimulus or shear stress promotes cell growth inhibition, metabolic changes in terms of intracellular bioenergetic pathway (aerobic or anaerobic), the activation of a number of cellular mechanosensors that transduce physical stimuli into biochemical signals, and triggers cellular differentiation (Neuhaus et al., 2006; Cucullo et al., 2008; Cucullo et al., 2013). Therefore, this system takes advantage of the exposure to variable and desired levels of pressure and/or shear stress in a pulsatile mode of pumping in comparison to some rotation-based flow devices, where shear stress gradients appear, and static cell cultures without flow perfusion (Neuhaus et al., 2006; Santaguida et al., 2006). Additionally, the HF perfusion bioreactor contains accessible ports to facilitate the cell seeding and sampling, as well as multiple electrodes for TEER measurement, which can be optionally added to indirectly assess the BBB reconstruction. Finally, the DIV-BBB system is placed in an incubator with a 95% of humidified atmosphere and 5% of CO_2_ at 37°C. The CO_2_ is needed as part of the media buffer system to regulate the pH and the specific incubator conditions are required to avoid cell cultures desiccation and optimal environment for cell growth (Janigro et al., 2000).

Interestingly, Santaguida et al. (2006) highlighted the significant increase of TEER values (651 ± 27 Ω cm^2^) when BAEC and C6 cells were cocultured in DIV-BBB systems (Figure 5B) in comparison to Transwell models (57 ± 3 Ω cm^2^, Figure 3B). Moreover, the aerobic metabolic pathway of the cells was reflected by the 1:1 ratio of glucose consumption and lactate production (Figure 5C) by the consequent improved oxygen supply to the cells of DIV-BBB systems. In addition, other works have demonstrated the efficiency of these DIV-BBB models *in vitro* BBB reconstruction by reaching TEER values as high as 1,200 Ω cm^2^ in the case of using human origin cocultures of endothelial cells and astrocytes (Cucullo et al., 2007; Cucullo et al., 2008), emerging the DIV-BBB systems as good candidates for the development of reliable *in vitro* BBB models and being its development highly recommended (Neuhaus, 2021). Thus, it can be claimed that in terms of biological relevance, 3D DIV-BBB culture systems are superior to 2D Transwell coculture systems.

## 3 Hollow fiber membranes as cell support in DIV-BBB systems

The role of HF membranes is key for the engineered *in vitro* DIV-BBB models. HFs must have the adequate morphological and mechanical features to simulate the ECM and to provide physicochemical cues for modulating cellular fate and differentiation. It is known that *in vitro* 2D static cell cultures, a layer of more than 100 µm could not be supported because of the restriction of diffusive mass transfer as happens in the development of 3D engineered organs or tissues (Eghbali et al., 2016). In this regard, the HFs actuate as an artificial vascular network in the DIV-BBB models avoiding hypoxic and necrotic conditions and ensuring constant supply of nutrients to the cells (Diban and Stamatialis, 2014). Moreover, the high and interconnected porosity of the HF walls protects cells from shear stress and is required to provide cell-to-cell contact in cocultures (Janke et al., 2013; Morelli et al., 2017a; Morelli et al., 2021). Therefore, the polymer selection and the tuning of the morphological and physicochemical properties of the HFs are critical to ensure mass transfer by diffusion and/or convection of the perfused culture medium and intercommunication *via* regulatory factors between endothelial cells and astrocytes co-cultures in the luminal and abluminal side, and finally, to recapitulate *in vivo* BBB functionality. Thus, Table 1 collects an overview of the different characteristics of the hollow fiber membranes used in DIV-BBB flow-based models so far.

**TABLE 1:** Overview and characteristics of the hollow fiber membranes used in DIV-BBB flow-based models.

Material	Processing technique	Morphological features and system dimensions		Surface coating	Refs.
Polypropylene	Commercial (CELLMAX® QUAD, Cellco)	∅_int_ = 75 µm; δ = 225 µm; p_s_ = 0.5 µm; n = 50	V_lum_ = 0.5 ml; S_lum_ = 70 cm^2^; V_ablum_ = 1.4 ml; S_ablum_ = 100 cm^2^	ProNectin^TM^ F; Fibronectin, poly-D-lysine (3 µg cm^-2^)	Stanness et al. (1997), Krizanac-Bengez et al. (2003), Parkinson et al. (2003)
Polypropylene	Commercial (CELLMAX® QUAD, Cellco)	∅_int_ = 480 µm; δ = 150 µm; p_s_ = 0.5 µm; n = 50	V_ablum_ = 1.5 ml	ProNectin^TM^ F; Fibronectin-like protein	Stanness et al. (1999), McAllister et al. (2001)
Polypropylene	Commercial (Spectrum Laboratories, Inc.)	∅_int_ = 330 µm; δ = 150 µm; p_s_ = 0.5 µm; n = 50 - 322	l = 13 cm; V_lum_ = 0.01 ml; S_lum_ = 1.3 cm^2^; V_ablum_ = 1.4 ml; S_ablum_ = 2.6 cm^2^	ProNectin^TM^ F; Fibronectin, poly-D-lysine (3 µg cm^-2^)	Neuhaus et al. (2006), Cucullo et al. (2007), Cucullo et al. (2008)
Polypropylene	Commercial (Accurel® Q3/2, Membrana)	∅_int_ = 600 ± 90 µm; δ = 200 ± 45 µm; p_s_ = 0.64 µm; n = 12; ε = 75%	l = 8.6 cm; V_lum_ = 0.024 ml; S_lum_ = 1.62 cm^2^; S_ablum_ = 1.32 cm^2^	ProNectin^TM^ F, fibronectin (30 µg ml^-1^); Fibronectin, poly-D-lysine (3 µg cm^-2^)	Cucullo et al. (2002), Cucullo et al. (2013)
Polypropylene	Commercial (Accurel® Q3/2, Membrana)	∅_int_ = 600 µm; δ = 200 µm; p_s_ = 0.64 µm; n = 19; ε = 75%	V_lum_ = 0.202 cm^3^; S_lum_ = 0.71 cm^2^; V_ablum_ = 1.15 cm^3^; S_ablum_ = 1.19 cm^2^	Fibronectin, poly-D-lysine (3 µg cm^-2^)	Santaguida et al. (2006)
Polypropylene	Commercial (Accurel® Q3/2, Membrana)	∅_int_ = 600 ± 90 µm; δ = 200 ± 45 µm; p_s_ = 2–4 µm; n = 11; ε > 75%	l = 8.6 cm; V_lum_ = 0.024 ml; S_lum_ = 1.62 cm^2^; S_ablum_ = 1.32 cm^2^	Fibronectin, poly-D-lysine (3 µg cm^-2^)	Cucullo et al. (2011)
Polysulfone	Wet spinning	∅_int_ = 640 µm; δ = 45 µm; p_s_ = 0.34 ± 0.16 µm	l = 5.8 cm; S_lum_ = 16.3 cm^2^	Fibronectin (2 µg cm^-2^)	Ciechanowska et al. (2016)
Polyacrylonitrile	Dry-jet wet spinning	∅_int_ = 568 ± 26 µm; δ = 138 ± 14 µm; MW_cut-off_ = 490 kDa	V_bioreactor_ = 1.7 ml; S_membrane_ = 23 cm^2^; K_W_ = 146 L m^-2^ h^-1^ bar^-1^	Poly-L-lysine (40 µg cm^-2^)	Morelli et al. (2016)
Poly(L-lactic acid)	Coaxial electrospinning	∅_int_ = 75 ± 8 µm; δ = 3.4 ± 0.7 µm; p_s_ = 0.36 ± 0.14 µm; ε = 46 ± 7%	V_bioreactor_ = 2.4 ml; S_bioreactor_ = 7.12 cm^2^; K_W_ > 580 L m^-2^ h^-1^ bar^-1^	Without surface coating	Morelli et al. (2017b), Morelli et al. (2019)
Polyvinylidene fluoride	Commercial (FiberCell Systems)	∅_int_ = 700 µm; δ = 280 µm; p_s_ = 0.1 µm	l = 4.35 cm; V_lum_ = 0.1 ml; V_ablum_ = 0.1 ml	Human fibronectin, (100 µg ml^-1^)	Moya et al. (2020)

∅ _int_, HF internal diameter; ε , HF porosity; δ , HF thickness; K_W_, hydraulic permeance; l, HF length; MW_cut-off_, molecular weight; n, number of HFs in the cartridge; p_s_, HF surface pore size; S_ablum_, abluminal HF surface area; S_lum_, luminal HF surface area; S_membrane_, membrane surface area; V_ablum_, volume of the abluminal side of the cartridge; V_bioreactor_, bioreactor volume; V_lum_, volume of the luminal side of the cartridge.

Table 2 shows the experimental conditions and major findings for cell culture models developed on the DIV-BBB flow-based systems presented in Table 1. It presents the assessment of the metabolism behavior of cells examined by nutrients consumption (i.e., glucose), and metabolites production (i.e., lactate), the typical molecular paracellular tracers to study the permeability across the BBB, and the intracarotid infusion of mannitol, a cell-impermeable and non-toxic polyalcohol that reversibly damages the BBB *in vivo*, to disrupt the BBB and to enable the passage of chemotherapeutic medicines (e.g., methotrexate) (Marchi et al., 2010; Cucullo et al., 2013). Furthermore, the permeability and TEER values are collected as well as the conditions of shear stress used in the cell cocultures.

**TABLE 2:** Cell culture conditions and measured variables in the *in vitro* studies performed in the DIV-BBB flow-based models.

Cell culture conditions	Biological parameters monitored	Permeability (cm s^−1^)	TEER (Ω cm^2^)	Shear stress (dyne cm^−2^)	Refs.
BAEC + C6, Seeding density = 1–2·10^7^ cells^;^ Culture time = 21 days	Cell morphology, pharmacological studies, K^+^ asymmetry, glucose consumption	Theophylline = 1.88·10^−6^; Sucrose = 8.8·10^−8^; Morphine = 5·10^−8^; Aspartate, mannitol = na	736 ± 38	1	Stanness et al. (1997)
RBMEC + RBA + B14, Seeding density = 2·10^6^ cells, Culture time > 20 days	Cell morphology, neuronal uptake of serotonin	Sucrose = 2.2·10^−6^; D-Aspartate = 2.1·10^−6^; L-Aspartate = 9.4·10^−6^	—	—	Stanness et al. (1999)
BAEC + RBA, Seeding density = na, Culture time = 21 days	Cell morphology, assessment of GLUT-1 asymmetry, glucose consumption	Sucrose = 7.6·10^−6^	—	1–5	McAllister et al. (2001)
BAEC + C6, Seeding density = 1.5–2·10^6^ cells, Culture time = 27 days	Glucose consumption, lactate production	—	500	Q = 4 ml min^−1^	Cucullo et al. (2002)
RBMEC + RBA, Seeding density = 5·10^6^ cells, Culture time > 14 days	Glucose consumption, lactate production, p_O2_/p_CO2_, NO and cytokines measurement	—	> 400	4	Krizanac-Bengez et al. (2003)
RBMEC + RBA, Seeding density = 5·10^6^ cells, Culture time > 14 days	Cell morphology, [^3^H] adenosine uptake and metabolism	FITC-albumin, sucrose, adenosine = na	—	4	Parkinson et al. (2003)
BAEC + C6, Seeding density = 1.5-2·10^6^ cells, Culture time > 7 days	Glucose consumption, lactate production, BBB disruption	Sucrose = 5.3·10^−6^; Phenytoin = 1.5·10^−5^	651 ± 27	4	Santaguida et al. (2006)
PBMEC/C1^−2^ + C6, Seeding density = 5–10·10^7^ cells, Culture time > 21 days	Glucose consumption, lactate production, FD4 standard marker	Nitrazepam = 9.9·10^−6^; Diazepam = 7·10^−6^; Flurazepam = 2.5·10^−5^	—	2.7–3.9	Neuhaus et al. (2006)
HBMEC + HA, Seeding density = 4–6·10^6^ cells, Culture time = 28 days	Glucose consumption, lactate production, BBB disruption	Sucrose = 3.9·10^−7^; Phenytoin = 1.7·10^−5^; Diazepam = 4.8·10^−3^	> 1100	4	Cucullo et al. (2007)
Three co-cultures: HBMEC + HA, HUVEC + HA, AVM-EC + HA, Seeding density = 4–6·10^6^ cells, Culture time > 21 days	Glucose consumption, lactate production, interleukin and MMP activity, BBB disruption	Sucrose = 3·10^−7^; Phenytoin = 8.4·10^−6^; Diazepam = 2·10^−3^; Sucrose = 1.2·10^−6^; Phenytoin = 1.6·10^−5^; Diazepam = 2·10^−3^; Sucrose = 1.1·10^−6^; Phenytoin = 6.1·10^−5;^ Diazepam = 2·10^−3^	1200 except for HUVEC + HA	4	Cucullo et al. (2008)
HBMEC + HA, Seeding density = 1.5–2·10^6^ cells, Culture time = 21 days	Glucose consumption, lactate production, cytokines and MMP activity, BBB disruption	Sucrose = 3.16·10^−6^; Phenytoin = 6.75·10^−5^; Diazepam = 6.88·10^−3^	524	4	Cucullo et al. (2011)
Two systems: HBMEC + HA (capillary) HBVSMC + HBMEC (venule), Seeding density = 1.5–2·10^6^ cells, Culture time > 21 days	Glucose consumption, lactate production, pressure analysis, BBB disruption	Sucrose = 4.4·10^−7^; Phenytoin = 2.6·10^−5^; Diazepam = 4·10^−3^; Sucrose = 4.9·10^−5^; Phenytoin = 7.2·10^−5^; Diazepam = 4.1·10^−3^	> 700 capillaries, < 400 venules	Capillary = 16.3; Venule = 2.6	Cucullo et al. (2013)
HUVECs Seeding density = 6·10^4^ cells cm^−2^, Culture time > 3 days	Glucose consumption, lactate production, p_O2_, vWF and sICAM-1 markers assessment	—	—	6.6	Ciechanowska et al. (2016)
SH-SY5Y Seeding density = 1.5·10^4^ cells cm^−2^, Culture time > 14 days	Glucose consumption, oxygen uptake rate, crocin treatment, β-Amyloid toxicity, ROS measurement	—	—	Q = 0.96 ml min^−1^	Morelli et al. (2016)
SH-SY5Y Seeding density = 5·10^4^ cells cm^−2^, Culture time > 13 days	Glucose consumption, neuronal markers evaluation and guiding, BDNF released	K_glucose_ = 980 L m^−2^ h^−1^ bar^−1^; K_medium_ = 940 L m^-2^ h^−1^ bar^−1^; K_glycitein_ = 760 L m^−2^ h^−1^ bar^−1^	—	Q = 0.37 ml min^−1^	Morelli et al. (2017b), Morelli et al. (2019)
hCMEC/D3 + HA Seeding density = 1–5·10^6^ cells ml^−1^, Culture time > 7 days	Cell morphology, paracellular and transcellular modulation, flow alignment, NO production	Dextran (7 days) = 8.3·10^−6^; Dextran (14 days) = 3.3·10^−6^	—	3	Moya et al. (2020)

AVM-EC, arteriovenous malformations endothelial cells; BDNF, brain-derived neurotrophic factor; B14, immortalized rat neuronal cell line; FD4, FITC-dextran 4000; HA, human astrocytes; HBVSMC, human brain vascular smooth muscle cells; HUVECs, human umbilical vein endothelial cells; K_medium_, culture medium permeance; K_glucose_, glucose permeance; K_glycitein_, glycitein permeance; MMP, matrix metalloproteinase; na, not available; NO, nitric oxide; PBMEC/C1-2, porcine brain microvascular endothelial cell line; p_CO2_, partial pressure of carbon dioxide; p_O2_, partial pressure of oxygen; Q, flowrate; RBA, rat brain astrocytes; RBMEC, rat brain microvascular endothelial cells; ROS, reactive oxygen species; SH-SY5Y, human neuroblastoma cells.

As shown in Table 1, DIV-BBB HF models were first developed in 1997 by Stanness et al. (1997), who engineered a dynamic and tridimensional BBB model using HFs made of polypropylene (PP) with a surface pore size of 0.5 µm. The cell coculture of BAECs and C6 cells exhibited similar features to the *in vivo* BBB conditions, including a firm barrier to some ions and proteins, the formation of tight junctions, and high TEER values (Table 2). Albeit the study supported the hypothesis that endothelial-glial cell cocultures were required to induce a viable BBB, it did not reproduce all the *in-situ* BBB properties. Since then, huge efforts have been made to improve DIV-BBB models, mainly focused in the source of cell lines used (Banerjee et al., 2016; Bhalerao et al., 2020), but less common in the characteristics of the perfusion bioreactor system and the hollow fiber membranes used as scaffold for cell support (Williams-Medina et al., 2021; Jagtiani et al., 2022).

### 3.1 Membrane materials and processing techniques

Regarding the HFs nature and materials, it is important to mention that the hydrophilic/hydrophobic character of the polymeric HFs and the surface wettability can lead to determine the fate of cell anchorage and the proliferation by changing the degree of contact between cellular receptors and the physiologic environment (Mantecón-Oria et al., 2022). The traditional HF materials most widely employed in commercial DIV-BBB systems are PP (Table 1), which hydrophobicity nature inhibits the cellular adhesion capacity since they do not allow the adsorption of proteins and other biomolecules (Machado, 2012; Morelli et al., 2021). Polysulfone (PS) HFs synthetized by wet spinning have also been reported for this application (Ciechanowska et al., 2016). They incorporated PS HFs in a tailor-made perfusion bioreactor where HUVEC cells were cultured to recreate *in vitro* human blood vessels and to study different pathophysiological mechanisms (Table 2). The PS offers good chemical resistance in physiological conditions, and acts as semi-permeable membrane with adaptable porosity allowing high transmembrane mass transport (Dufresne et al., 2013). However, PS is also a hydrophobic polymer with similar behavior for cell adhesion and spreading as PP. Morelli et al. (2016) developed polyacrylonitrile (PAN) HF membranes using the dry-jet wet spinning technique with polyvinylpyrrolidone (PVP) as pore-former. PAN HFs exhibited high mechanical resistance and hydraulic permeability providing an improvement in the mass transfer and maintaining SH-SY5Y cells survival in the membrane bioreactor for the *in vitro* reconstruction of a neuronal network (Table 2). In this work, the water contact angle of 67.5° indicated the hydrophilic character of these PAN HFs. It was reported that the optimum contact angle for cell attachment is ∼64° with a decrease in cell adhesion on very hydrophilic or very hydrophobic surfaces (Bajaj et al., 2014). Morelli et al. (2017b), Morelli et al. (2019) also reported the successful incorporation of poly (L-lactic acid) (PLLA) micro-hollow fiber membranes on a perfusion bioreactor system mimicking the CNS microenvironment. PLLA was selected due to its biodegradable nature being one of the most promising biopolymers for neural tissue engineering as well as is approved for human clinical applications (Yang et al., 2005; Saini et al., 2016). More recently, Moya et al. (2020) reported the use of commercial polyvinylidene fluoride (PVDF) hollow fibers assembled in a two molded PDMS structures to *in vitro* BBB formation (Table 1). The high electrocapacitive feature of PVDF has the potential to convert mechanical, thermal, or magnetic signals into electrical ones interesting for biomedical applications (Costa et al., 2013; Martins et al., 2014). However, the hydrophobic character of PVDF is also limiting the adhesion of cells. To favor the cell adhesion over hydrophobic HFs, authors coated the HF internal surface with ECM molecules, such as fibronectin (Ciechanowska et al., 2016), and externally with poly-D-lysine (PDL) or poly-L-lysine (PLL) (Morelli et al., 2016) to favor endothelial adhesion and neuronal or astrocytic growth, respectively (Table 1). The employment of these adhesive molecules implies an increment in the cost and experimental complexity associated with the development of these models. Outstandingly, Morelli et al. (2017b), Morelli et al. (2019) did not use any surface coating over the PLLA HFs for promoting the growth and differentiation of SH-SY5Y cells toward a neuronal phenotype (Table 1 and Table 2).

**FIGURE 6:**
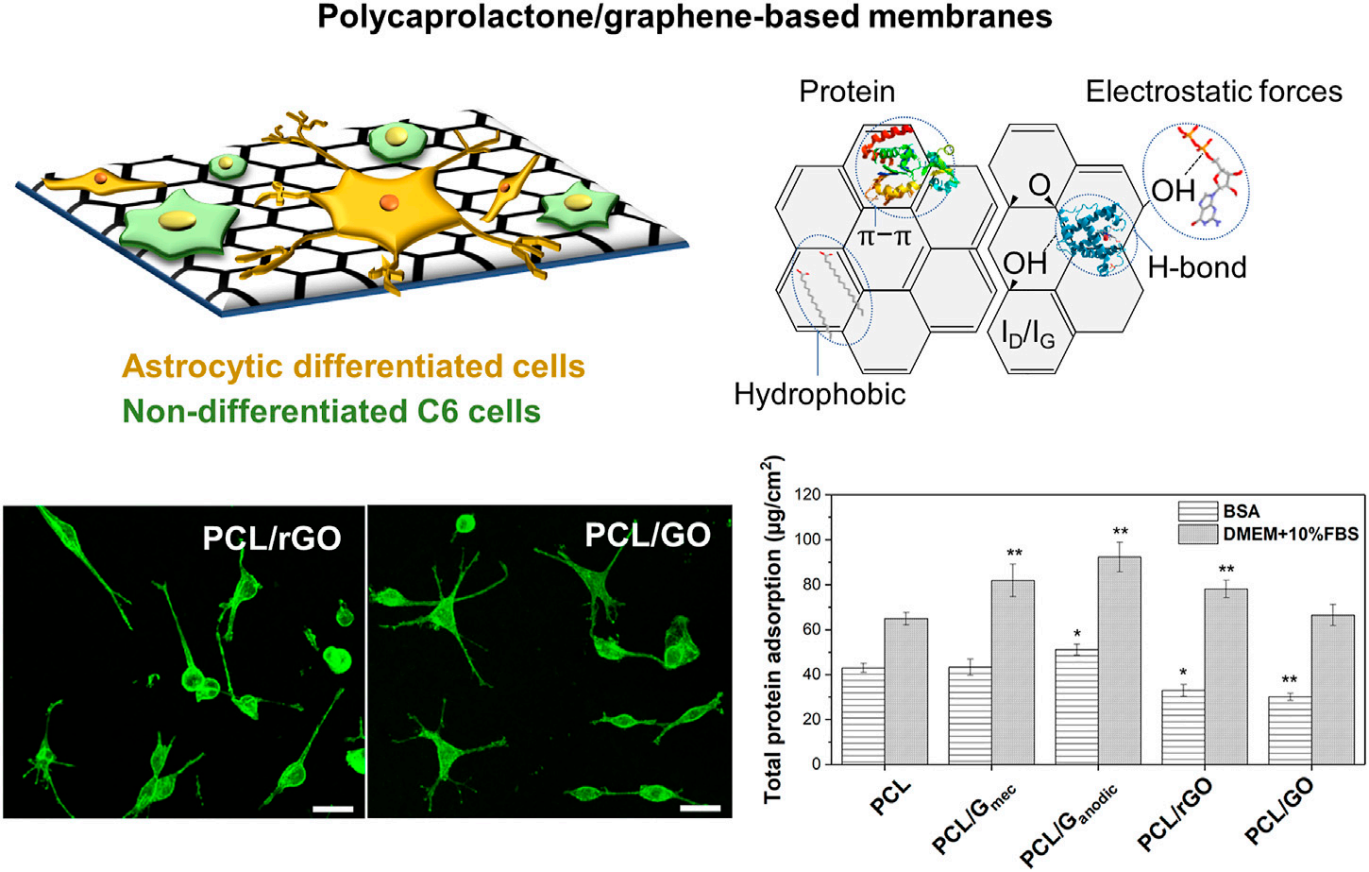
Defective graphene-based nanomaterials uniformly dispersed in PCL flat membrane surface significantly favored cell anchorage and astrocytic differentiation *via* chemical structural defects and protein selective adsorption. Confocal images depict the morphological analysis of C6 cells stained with Phalloidin-FITC and differentiated towards astrocytes on PCL/rGO and PCL/GO flat membranes. The total protein adsorption of BSA model protein and cell culture media (DMEM +10% FBS) on PCL and PCL/GBN flat membranes is also presented. Statistical significance with respect to PCL (*p < 0.05; **p < 0.01). Reprinted with permission from (Mantecón-Oria et al., 2022), Creative Commons Attribution 4.0 International License.

The role of the polymer materials can also go beyond cell adhesion interactions. Interestingly, the use of additives, polymers blends, and surface functionalization of the polymeric HFs has received increasing attention with the purpose of improving its bioactivity for tissue engineering applications (Stratton et al., 2016; Tolou et al., 2021; Yang et al., 2022). Among these alternatives, graphene-based nanomaterials (GBNs) have been explored in drug delivery, diagnostics, cancer therapy and tissue engineering applications (Shin et al., 2016; Kumar et al., 2021). In particular, the incorporation of GBNs has demonstrated outstanding results for neural and nerve regeneration having the capacity of inducing cellular differentiation (Sánchez-González et al., 2018a; Gupta et al., 2019; Girão et al., 2020), as well as changing the electrical and mechanical properties, and the degradation rates of the composite scaffolds (Sánchez-González et al., 2018b). Some works report on the higher electrical properties of these nanomaterials and the physicochemical features as the characteristics that could enhance this cellular differentiation (Sánchez-González et al., 2018a; Luong-Van et al., 2020). Meanwhile, other works reported the enhanced adsorption of PLL proteins due to the presence of GBNs on polycaprolactone (PCL)-gelatin electrospun nanofibers (Girão et al., 2020) causing and improvement in neural cell differentiation. Similarly, Mantecón-Oria et al. (2022) recently pointed to the chemical structural defects in reduced graphene oxide (rGO) and graphene oxide (GO) nanomaterials, and the protein adsorption mechanisms as the most plausible cause conferring distinctive properties to PCL/GBN membranes for the promotion of astrocytic differentiation (Figure 6). Remarkably, this study suggested that the lower adsorption of bovine serum albumin (BSA) globular protein on PCL/rGO and PCL/GO flat membranes could enhance the adsorption of other proteins such as fibronectin, laminin or vitronectin promoting a direct linkage between the cellular receptors to the membrane surface and triggering astrocytic differentiation. Other works reinforce this hypothesis showing the protein adsorption as a competitive process on biomaterials surface (Kumar and Parekh, 2020). Therefore, a critical analysis of the type of protein corona on different membrane substrates can guide the design of novel and functional scaffolds.

It is well known that the mechanical properties of the scaffolds significantly impact on cell activity and maintenance of tensional homeostasis, on driving cell–material interactions, and on regulating and guiding the formation of new tissues (Morelli et al., 2015; Salerno et al., 2020). Regarding the mechanical properties’ requirements for engineered scaffolds, soft biomaterials such as hydrogels could favor the organization and function of tissues, particularly neural ones, since its values of stiffness are in the scale of kPa, which matches better with the mechanical properties of the native tissues (Vedadghavami et al., 2017; Li et al., 2018). In comparison, the reported polymer HFs used in DIV-BBB models (Table 1) exhibit mechanical properties in the range of MPa. For instance, PLLA HFs had a Young modulus of 78.8 ± 4.7 MPa and an ultimate tensile strength of 1.8 ± 0.1 MPa (Morelli et al., 2017b; Morelli et al., 2019) whereas these values were 17.34 ± 0.79 MPa and 1.65 ± 0.03 MPa for PCL HFs (Mantecón-Oria et al., 2020), and 123.7 ± 19.7 MPa and 4.38 ± 0.15 MPa for PP HFs, respectively (Yan et al., 2019). Nevertheless, they provide more adequate structural stability and slow degradation rates preserving the mechanical properties *in vitro* over time than hydrogels (Zadpoor, 2015).

**FIGURE 7:**
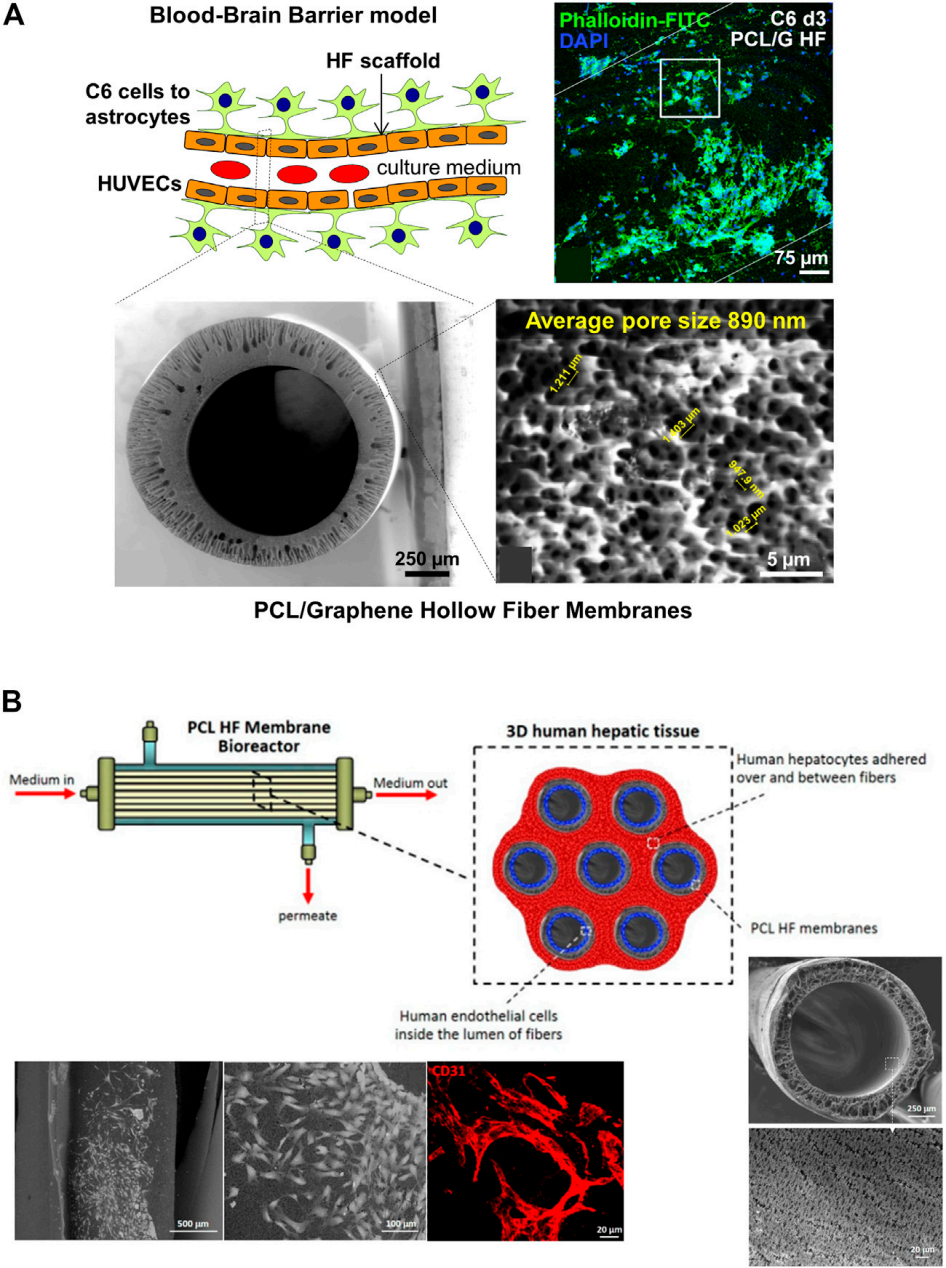
**(A)** Schematic representation of an ideal BBB model considering the differentiation of C6 cells towards astrocytes in the outer surface of the HF and the seeding of HUVECs in the lumen. The scheme depicted the cross-section and outer surface ESEM images of the PCL/G HFs with an average surface pore size of 0.89 µm. The representative confocal image shows the study of astrocyte differentiation for 72 h on PCL/G scaffolds where cells reduced their nuclear size and presented numerous cytoplasmic projections (Mantecón-Oria et al., 2020). **(B)** Scheme of a 3D human hepatic tissue model in a HF perfusion bioreactor by culturing human hepatocytes over and between a bundle of seven PCL HFs, and endothelial cells cultured in the lumen of the HFs. Scanning electron microscopy (SEM) images show the cross-section and lumen surface of the PCL HF membranes as well as the HUVEC cells cultured in the lumen of the HFs after 18 days. The confocal image shows the HUVEC differentiation in elongated cells and tube- and ring-like structures resembling capillary features, and expressing the CD31 in red (Salerno et al., 2020).

With respect to the processing of the materials, the HFs for DIV-BBB models are mostly synthetized using melt (such as commercial PP HFs) and wet spinning. For instance, Mantecón-Oria et al. (2020) developed a polymeric composite membrane of PCL and a multilayered graphene produced by mechanical exfoliation method (G) to obtain PCL/G hollow fibers by phase inversion (Figure 7A). During the membrane synthesis, the presence of graphene enlarged the pore size up to 0.89 ± 0.08 µm, a feature that significantly enlarged water permeability, and the electrical conductivity of PCL/G HFs compared to PCL HFs. It is of particular interest that in the PCL/G HFs the cell adhesion was relatively satisfactory without applying any surface coating despite the hydrophobic character of PCL. The biocompatibility assay with HUVEC and C6 cells revealed that PCL/G HFs significantly increased C6 cells adhesion and differentiation towards astrocytes while produced a cytotoxic effect on the endothelial cell line. These results agree with previous studies that demonstrated the large integration of HUVEC cells with the internal surface of PCL HFs forming vascular-like structures for the fabrication of a vascularized human liver tissue (Figure 7B) (Salerno et al., 2020). Similarly, the development of poly (lactic-co-glycolic acid) (PLGA) blends with PCL was previously presented as potential scaffolds for the development of small-caliber vascular grafts (Diban et al.,2013a; Diban et al., 2013b). Therefore, it was deemed that the HFs should be designed as a dual-layer HF consisting of a PCL/G layer in the outer surface for C6 culture and a PCL substrate in the lumen for endothelial cells culture with the purpose of being incorporated in DIV-BBB flow-based hollow fiber models.

It has been extensively established that topography and roughness can provide different physical and chemical cues to influence cellular responses (Guo et al., 2017; Lee et al., 2018; Palmieri et al., 2020). In this regard, electrospinning techniques result as one of the better options for creating scaffolds that imitate the ECM simulating the *in vivo* microenvironment and giving topographical and biophysical cues that regulate cellular morphogenesis (Park et al., 2016; Guo et al., 2019). Among the investigations made on this topic, Morelli et al. (2017b), Morelli et al. (2019) synthetized PLLA polymeric membranes by coaxial electrospinning with an external pore size of 0.36 ± 0.14 µm and a porosity of 46 ± 7%. This membrane configuration provided guidance cues to direct cell orientation and enhanced neural cell differentiation.

Furthermore, some 3D printing techniques are emerging using different natural and thermoplastic polymers and composites, to obtain higher mechanical resistance and functionality on BBB models (Bhalerao et al., 2020; Jagtiani et al., 2022). However so far, the different printing techniques have only been utilized in microfluidic devices. For instance, a porous PCL/PLGA vasculature network was recently synthetized by freeze-coating to explore drug BBB toxicology and permeability, and cell interactions (Yue et al., 2020). Nevertheless, the TEER of the reconstructed endothelial layer is relatively low (40–120 Ω cm^2^) compared to *in vivo* values.

Finally, the intrinsic lack of transparency of the commercial HFs limits the intraluminal observation of the ECs making impossible to analyze the cell morphology and phenotypic changes *in situ* at real time (Machado, 2012). Trying to improve the commercial HF membrane opacity, some works introduced the HFs with cultured cells in a mineral oil for a few minutes, then the hollow fibers became transparent and could be visualized by confocal or light microscopy (Williams-Medina et al., 2021). Similarly, (Zakharova et al., 2021) reported the fabrication of porous PDMS membranes for enabling live-cell imaging and phase-contrast microscopy without the need of fluorescent markers whereas (Moya et al., 2020) dipped the PVDF HFs in PDMS to achieve the optical transparency. Nevertheless, the PDMS impregnated the pores of the HFs and did not allow the passage of nutrients and the interconnectivity between the cells, so it is not useful to obtain a reliable *in vitro* BBB model. Thus, increasing attempts are being made to automate cell profiling by microscope assessment, a non-invasive method to track the development of the *in vitro* BBB models’ progress and enable the identification of important biomarkers inside and on the surface of the HFs.

### 3.2 Membrane morphology

Referring to morphological features, a large pore size in combination with a high porosity and a low thickness of the HF membranes are the most important parameters to promote successful physical and biochemical cell interactions between abluminal and luminal side of the hollow fiber perfusion bioreactor. Ideally, these characteristics should suit the cells cocultured physiological dimensions without compromising the HFs mechanical properties.

Regarding to the thickness of the membranes, the values indicated in Table 1 ranged from 150 to 280 µm for commercial PP and PVDF HFs, and are much larger than the values reported for *in vivo* capillary blood vessels, which have a wall thickness of 1 µm (Müller et al., 2008). The coaxial electrospun membranes synthetized by Morelli et al. (2017b); Morelli et al. (2019) are outstanding innovations because they achieved a reduction in the internal lumen diameter up to 75 μm, and a wall thickness of only 3.4 ± 0.7 µm (Table 1). This microtube conformation offered not only indirect perfusion to SH-SY5Y cells in the laminar flow regime promoting long-term growth and neuronal orientation and differentiation (Morelli et al., 2017b), but also a successful tool as investigational platform to screen new molecules and delay the progression of neurological disorders (Morelli et al., 2019). Similar results were proposed by Ciechanowska et al. (2016) obtaining a wall thickness of 45 µm in the HFs synthetized by wet spinning (Table 1). This work evaluated the metabolic changes by applying high shear stress in the lumen of the HFs obtaining that HUVEC cells consumed 1.5 times more glucose and produced 2.3 times more lactate in comparison to static cell cultures (Table 2), and the partial pressure of oxygen dissolved in the culture medium was close to the noted in the arterial blood *in vivo*. These works explored the ability of small HFs to promote neuronal and endothelial cell differentiation, but they did not study the effect of membrane thinning and did not perform cell cocultures for *in vitro* BBB studies. It is well-known that the astroglia release short-lived soluble growth factors that would not be able to reach the endothelial cell monolayer if it was seeded on a membrane thickness of more than 100 µm (Banerjee et al., 2016; Zidarič et al., 2022). Furthermore, large membrane thickness prevents the direct contact between cells cocultured if considering that the astroglia projections are around 37 and 98 µm in length, in rodents and humans, respectively (Oberheim et al., 2009). Overall, a HF membrane thickness lower than 50 µm will be required to promote cell interactions in cocultures.

The pore size of the HFs must permit the permeability of paracellular markers, the exchange of cell-secreted growth factors, the transport of different molecular size of drugs, nutrients, and cell-to-cell contact. As shown in Table 1, most of the works reported a transcapillary pore size in the HFs below 0.5 µm. In particular, commercial PP HFs with average pore size of 0.5 µm have been most widely employed in DIV-BBB systems as an attempt to favor the exchange of oxygen and carbon dioxide enhancing cell oxygenation and enabling the permeation of solutes, some proteins and the culture medium (Janigro et al., 2000; Di and Kerns, 2015). For Transwell models, it has been demonstrated that the area, pore size and composition of the membrane inserts used can alter the *in vitro* barrier integrity (Prashanth et al., 2021). Remarkably, Stone et al. (2019) compared 0.4 and 3 µm pore size PET membrane inserts (Corning Inc.) coated with collagen and found higher TEER values with the larger one as well as an improvement in the connection and communication between endothelial cells, astrocytes and pericytes, which resulted in greater barrier integrity. Additionally, some studies performed in Transwell systems evidenced that the astroglia projections of cells cultured on flat membranes with 0.4 µm pore size blocked the pores and prevented the physical contact of astrocyte foot processes with the ECs as well as the passage of soluble factors (Shayan et al., 2011; Banerjee et al., 2016). Moreover, several research groups used 1 μm pore size in Transwell membranes as they were suitable for permeability studies (Kuo and Lu, 2011; Neuhaus et al., 2011). Consequently, the formation of bigger pores in the HFs is mandatory to enable the physical contact between the astrocytic foot-processes and the endothelial cells across the fiber wall as well as the permeation of critical compounds. However, high pore size may produce undesirable cell extravasation across the compartments as was reported by Wuest et al. (2013) where pores of 3 μm and 8 μm in Transwell membrane inserts promoted the migration of cells in the membrane wall, building a second monolayer of endothelial cells, whereas with a 1 μm of pore diameter cells were not able to migrate through the pores. In DIV-BBB HF models, Cucullo et al. (2011) increased the transcapillary pores of a commercial PP HFs from 0.5 to 2–4 µm by mechanical piercing with a density of 100 pores per cm^2^ in the outer surface of the HFs (Table 1), to study the immune cell traffic across the BBB. Although the higher pore size favor the extravasation of immune cells trough the BBB, the permeability of sucrose (3.16·10^−6^ cm s^−1^), phenytoin (6.75·10^−5^ cm s^−1^), and diazepam (6.88·10^−3^ cm s^−1^) (Table 2) was still higher than *in vivo* reported data (1.00·10^−7^ cm s^−1^ for sucrose, 1.08·10^−5^ cm s^−1^ for phenytoin, and 2.20·10^−4^ cm s^−1^ for diazepam, respectively) (Santaguida et al., 2006; Heymans et al., 2018). These results are congruent with optimum 1–2 µm pore size observed for Transwell studies to avoid endothelial cells extravasation. Additionally, the high HF thickness of 200 ± 45 µm could reduce the exchange of regulatory factors and the contact between the HBMEC and the HA cells cocultured making the cell barrier less restrictive.

### 3.3 BBB recapitulation and functional assessment related with HFs properties and system

Results presented in Table 2 for DIV-BBB HF models exhibit great variability in terms of the cell source employed, the assessment of cell morphology and its metabolism, the methodology used to measure substances permeability, TEER values, and shear stress. Some of the presented studies examined the long-term effects of drugs on BBB formation (Stanness et al., 1999; Neuhaus et al., 2006; Morelli et al., 2016), others studied the kinetics of transendothelial drugs trafficking (Stanness et al., 1997; Krizanac-Bengez et al., 2003; Cucullo et al., 2008; Cucullo et al., 2011; Ciechanowska et al., 2016; Moya et al., 2020), the BBB disruption by mannitol (Santaguida et al., 2006; Cucullo et al., 2007, 2013), or the cell differentiation and relationships between astrocytes and endothelial cells in the development of the BBB (McAllister et al., 2001; Cucullo et al., 2002; Parkinson et al., 2003; Morelli et al., 2017b; Morelli et al.,2019).

Glucose consumption and lactate production are usually monitored during the dynamic experiment as well as the microscopic study of the cell morphology (Table 2). Additionally, to evaluate the BBB functionality, some studies measured the permeability of some molecular tracers trough this barrier, e.g., sucrose, aspartate, phenytoin, or diazepam. However, many other studies analyzed the activity of specific chemicals and drugs in the DIV-BBB model. For example, Morelli et al. (2016), developed a successful neuronal *in vitro* model to test the effect of the carotenoid crocin on Aβ-mediated toxicity associated to Alzheimer’s disease, maintaining their functionality up to 2 weeks (Table 2). They studied the water filtration of PAN HFs with a wall thickness of 138 µm and molecular weight cut-off of 490 kDa (Table 1), showing a hydraulic permeance of 146 L m^−2^ h^−1^ bar^−1^ that permitted sufficient supply of nutrients in the extracapillary space by perfusion.

The HF perfusion bioreactor introduces shear stress mimicking physiological conditions and may potentially enhance the vascularization and oxygenation of 3D cell cultures (Wung et al., 2014; Morelli et al., 2021). Commonly, the shear stress applied in DIV-BBB hollow fiber models is 4 dyne cm^−2^ (Table 2) which emulates the circulation of blood pressure *in vivo* (Cucullo et al., 2013; He et al., 2014). By applying these flow conditions in the intraluminal space, TEER values between 400 and 1,200 Ω cm^2^ were recorded (Table 2) depending on the experimental conditions used.

Furthermore, the use of different types of cells of the BBB will be important to create physiologically realistic models that closely resemble the heterogeneous *in vivo* circumstances. Specifically, the highest TEER values (>1,100 Ω cm^2^) reported in Table 2 were obtained when cocultures of human endothelial and astrocytic cells were used, therefore imitating better the *in vivo* human BBB (Cucullo et al., 2007; Cucullo et al., 2008). Nevertheless, the DIV-BBB hollow fiber models collected in Table 2 still presented much lower TEER values than the 5,000 Ω cm^2^ reported for human BBB tissues (Banerjee et al., 2016; Srinivasan and Kolli, 2019).

After the critical analysis of the literature reporting DIV-BBB HF models, in view of the variability in the experimental procedures used and the diversity of the studies performed, comparing the results obtained and assessing the importance of each parameter is complicated. Nevertheless, analyzing Table 1 and Table 2 together, the high thickness and small pore size of the commercial HFs seem to explain to some extent the failure in realistically reproduce immune transmigration and transendothelial cell trafficking, as well as the low transport of regulatory factors, nutrients, and metabolites between ECs and astrocytes co-cultured.

## 4 SWOT analysis of DIV-BBB flow-based hollow fiber models

In recent years several efforts have been made to make progresses for a benchmark BBB model technology. Despite cell morphology and phenotype on *in vitro* 2D Transwell models significantly differ from the *in vivo* cells, it seems that the 2D coculture on Transwell systems is still the most widely *in vitro* model used nowadays due to the robustness, flexibility, and cost-effectiveness.

Meanwhile, the DIV-BBB models exhibit interesting strengths (Table 3) over Transwell systems. For instance, DIV-BBB models have significantly higher TEER values and lower permeability coefficients than Transwell models achieving an asymmetric distribution of glucose consumption similarly to that reported *in vivo* (Janke et al., 2013). It means that the tightness of the BBB model created by the DIV-BBB hollow fiber systems is more stringent than the static 2D models. This is generally associated with the capacity of these systems to closely mimic the anatomy and hemodynamic conditions of brain capillaries.

**TABLE 3:** Summary of the SWOT analysis to get DIV-BBB systems as commercial gold-standards.

Strengths	Weaknesses	Opportunities	Threats
Versatile system able to mimic *in vivo* conditions	High cell seeding density	Research and technology transfer capacity to produce advanced HF materials	Complex and time-consuming protocols of cell culture
Easy scalable system toward high-throughput devices	Difficulty for the on-line microscopy assessment of the luminal side		Insufficient biological matching between *in vivo* and *in vitro* DIV-BBB models
Low-cost fabrication, ease sampling	Non-reusable		
Long-term cell viability: multiple drug assays in a single DIV-BBB cartridge	Poor features of the HFs commercially available		

Additionally, DIV-BBB hollow fiber models are easily scalable, tunable, and low-cost. The synthesis of HFs is a well stablished technology at industrial scale with competitive costs. Besides, the tubular configuration of the HFs and its small diameter provides high compactness with large surface area to bioreactor volume ratio (30 cm^2^ cm^−3^) that allows high cell expansion densities (Diban and Stamatialis, 2014; Eghbali et al., 2016; Morelli et al., 2021), and facilitates the quantitative monitorization of pharmacokinetic studies (Cadwell and Whitford, 2017; Morelli et al., 2021). On the other hand, these systems can also be easily scale down towards high-throughput devices. Other strong point on DIV-BBB models is related to the ability to be used for a long-term once the BBB have been recapitulated. This capacity permits the study of administering different drugs and biochemical agents into the intraluminal or extraluminal compartments (Santaguida et al., 2006) avoiding the interexperimental variability over time. Finally, these dynamic *in vitro* systems can be also beneficial for other cell cultures and for research, e.g., predicting pharmacokinetics/pharmacodynamics targeted to infectious diseases (Tanudra, 2018), guiding hepatic differentiation of human mesenchymal stem cells (Piscioneri et al., 2018), mimicking skin vascularization (Perez Esteban et al., 2019), and recreating liver tissue (Salerno et al., 2020).

Despite all the above advantages of DIV-BBB flow-based hollow fiber systems, they are still far from becoming a commercial gold standard. This can be attributed to certain threats as: 1) the complex operation of these systems compared to 2D Transwell systems and 2) they do not yet match the *in vivo* BBB physiological functionality. Some of these threats are related with certain weaknesses (Table 3). For instance, DIV-BBB systems require culturing at tissue-like densities (>10^4^ cells cm^−2^) (Cucullo et al., 2002; Cadwell and Whitford, 2017) that implies long times for *in vitro* BBB recapitulation. It takes around 9–12 days to reach the TEER steady-state values compared to the 3–4 days needed in coculture Transwell models (Janigro et al., 2000; He et al., 2014). For the microscopic analysis, it is widely spread the use of optical glass or PC in the external shell of the perfusion bioreactor to direct visualize the cells cultured in the abluminal side. However, the cells cultured in the lumen of the HFs cannot be observed due to opacity of the polymeric membranes. Thus, it will be necessary to sacrifice the HF module of the perfusion bioreactor to perform cellular staining with specific markers and histological sectioning at the end of the experiment (Mahaffey, 2012). Consequently, the HF cartridges cannot be reused. Moreover, the commercial HFs currently used in these devices present low cell adhesion due to their hydrophobic properties, and small pore sizes and high wall thickness, which limits the exchange of regulatory factors and cell interactions between the characteristic BBB cell types cultured in the HFs (Morelli et al., 2016; 2017b).

### 4.1 Challenges and opportunities

During this work, it has been detected that the mismatching between native and DIV-modeled BBB could be attributed to: 1) a low transmembrane exchange of regulator factors by cocultured cells, 2) a low cell adhesion on the HFs, and 3) an inability of the HFs material to induce cell differentiation. These issues were mainly caused by the large thickness (>100 μm) and small pore size (<0.5 μm) of the commercial HF membranes currently employed in DIV-BBB models. Other challenges are related to: 1) the opacity of HFs that hampers the direct online monitorization of the BBB recapitulation by microscopic techniques, which must be estimated using indirect techniques as TEER or sacrificing the membrane module, and 2) the high experimental demand in terms of cell culture density and times required to recapitulate steady-state BBB *in vitro*.

These parameters are critical in the design of DIV-BBB models, which we consider as a research and technology transfer opportunity to foster the technology of DIV-BBB (Table 3). Therefore, the following guidelines are proposed below to help researchers to deal in the future with the above-mentioned limitations:

⁃ The use of biocompatible amorphous polymers or semi-crystalline polymers processed under controllable precipitation pathways to produce spherulites with size domains <0.4 µm to allow the light transmission through the membranes and therefore favoring their transparency.
⁃ Appealing to hydrophilic biopolymers such as PVP, polyethylene oxide (PEO), polyethylene glycol (PEG) or polyvinyl alcohol (PVA) (Erothu and Kumar, 2016) to benefit cell adhesion and protein adsorption, and the diffusion of small biological molecules in 3D DIV-BBB coculture models.
⁃ Producing thin HFs (<50 μm) by controlling the extrusion of the HFs to improve the light transmittance trough the membrane wall and facilitating the transport of regulation factors and cell interconnectivity.
⁃ The use of several strategies can be applied to optimize the porous morphology of the polymeric HFs that are traditionally prepared by wet phase inversion spinning technique to achieve effective pore sizes of 1–2 µm. For instance, the incorporation of pore formers (e.g., PVP, PEG) in the dope solution or additives (water, ethanol, organic solvents, hydrophilic nanofillers) to control the rate of exchange between the solvent and non-solvent of the phase separation system to enlarge the porosities.
⁃ The development of innovative bio-coextrusion spinning of hollow fiber membranes, as the next-generation 4D biofabrication technology.
⁃ Scaling down to HF microfluidic devices (high-throughput systems) for personalized medicine.
⁃ The use of experimentally validated simulation tools, i.e., computational fluid dynamics (CFD) software, to predict a larger number of parameters (e.g., glucose consumption and lactate production, transmembranal pressure, TEER values) reducing experimental costs and time.

After a SWOT (strengths, weaknesses, opportunities, and threats) analysis, the main strengths, weaknesses, opportunities, and threats of the DIV-BBB systems are collected in Table 3.

## 5 Conclusion

In the last decades the research on *in vitro* blood-brain barrier (BBB) models, essential in the study of neurodegenerative diseases and drug development, has advanced from 2D monolayer cultures to 3D dynamic *in vitro* (DIV)-BBB models. This work has focused on the DIV-BBB flow-based hollow fiber (HF) system as one of the currently available alternatives to create a suitable microenvironment and to offer a wide range of physiological cues that stimulates and facilitates the cellular response, enhancing the vascularization and oxygenation of 3D cell cultures. This model is the most realistic and reliable for recapitulating the BBB *in vitro* although it still yields far from *in vivo* BBB. To identify the strengths, weaknesses, opportunities, and threats of DIV-BBB systems, we have made an integral and critic retrospect of different BBB *in vitro* models with a particular focus on the HFs employed as scaffolds in the DIV-BBB flow-based HF systems. Different HFs properties were analyzed, e.g., the polymeric materials used, the morphological features, and other performing properties. Open research opportunities to get DIV-BBB models as a benchmark in the future cover from the improvement of morphological features, i.e., thickness and pore size, and transparency of the HFs to the optimal design of the HF cartridges with the help of key-enabling technologies such as the nanotechnology, and advanced manufacturing technologies, e.g., the microfluidics and 4D biofabrication.
